# Novel Insights in the Genomics of Anaplastic Thyroid Carcinoma: A Role for Cyclin-Dependent Kinase Inhibition?

**DOI:** 10.3390/cancers15184621

**Published:** 2023-09-18

**Authors:** Adam Stenman, Carl Christofer Juhlin

**Affiliations:** 1 Department of Molecular Medicine and Surgery, Karolinska Institutet, 17177 Stockholm, Sweden; adam.stenman@ki.se; 2 Department of Breast, Endocrine Tumors and Sarcoma, Karolinska University Hospital Solna, 17176 Stockholm, Sweden; 3 Department of Oncology-Pathology, Karolinska Institutet, 17164 Stockholm, Sweden; 4 Department of Pathology and Cancer Diagnostics, Karolinska University Hospital Solna, 17176 Stockholm, Sweden

Anaplastic thyroid carcinoma (ATC) stands as a rare but extraordinarily lethal tumor, marked by its limited treatment options. This dire prognosis is a consequence of the tumor’s remarkable invasive potential, prompting local infiltration of vital neck structures and rapid development of distant metastases. Moreover, ATCs display refractoriness to conventional therapeutic avenues like chemotherapy or external radiation [1]. The bulk of ATCs are thought to develop from pre-existing well-differentiated thyroid carcinoma (WDTC) as part of a dedifferentiation process, and this theory is supported by histological evidence of a WDTC in close conjunction to the ATC as well as phylogenetic evidence suggesting a shared clonal ancestry [2]. Hence, most ATCs harbor driver mutations regularly found in their WDTC counterparts, such as oncogenic *BRAF* mutations derived from papillary thyroid carcinoma (PTC) and *RAS* mutations from follicular thyroid carcinoma (FTC) [3].

Throughout history, a curative approach has only been viable for those patients harboring diminutive primary tumors that are confined solely within the thyroid gland itself. However, advances in our comprehension of the molecular genetic underpinnings of this tumor type, combined with the potential of targeted treatments utilizing tailored medications, have offered a glimmer of improvement in prognosis [4]. In certain remarkable cases, even the regression of disseminated disease has been observed. Notably, these instances have largely been attributed to the unearthing of the oncogenic *BRAF* V600E mutation, a genetic event occurring in a large subset of ATCs, thereby enabling treatment using BRAF inhibitors [5]. The notion that targeted therapy may offer prolonged patient survival highlights the pressing necessity for meticulous genetic mapping of this tumor variant to reveal further treatment targets.

The foundation of the existing genetic insights into ATC has been rooted in studies employing single-gene analyses and whole-exome sequencing. Nevertheless, research endeavors involving whole-genome sequencing (WGS) remain relatively scarce. The latter approach carries a suite of advantages, including the interrogation of the entirety of an individual’s genome, capturing variations in both coding and non-coding regions, allowing the identification of mutations occurring outside of exonic DNA, such as in regulatory regions and gene promoters. Moreover, large-scale genomic alterations can be more easily detected via WGS as the analysis also encompasses non-coding sequences that may be involved in complex deletions or duplications. Finally, WGS has the potential to provide unique insights into focal and genome-wide mutational distribution patterns that are lost when only the coding regions are studied.

In a recent study, researchers from Karolinska Institutet and Lund University in Sweden have embarked on a comprehensive exploration of ATC, utilizing a panoramic whole-genome outlook coupled with RNA sequencing [6]. This strategy not only unveiled the intricate tapestry of coding mutations, but also helped characterize complex chromosomal alterations and gene expression signatures. Beyond reaffirming the presence of genes conventionally implicated in thyroid cancer, the researchers have corroborated initial indications indicating that a substantial majority of these tumors exhibit deletions of the *CDKN2A/B* genes [7]. These genes encode three pivotal proteins (p14, p15 and p16) regulating the cell cycle, specifically governing the growth phase 1 (G1)-synthesis (S) transition by p15- and p16-mediated inhibition of cyclin-dependent kinase (CDK) 4/6 and cyclin D activities (Figure 1A). Furthermore, the p14 protein influences the entry into G1 through p21-mediated inhibition of CDK1 and cyclin B. Deletion of *CDKN2A/B* thus leads to the removal of this regulatory restraint, subsequently propelling cell cycle progression (Figure 1B). The intriguing facet of this genetic anomaly lies in the potential sensitivity of these tumors to CDK inhibitors—a class of drugs already sanctioned for treating hormone receptor-positive, *HER2*-negative, advanced breast cancer [8]. Considering the acute demand for personalized molecular interventions for this patient cohort, this discovery bears significant promise, warranting subsequent clinical investigations. Indeed, preclinical models suggest that CDK inhibitors may have anti-proliferative effects in ATC cell lines as well as in xenograft models [9].

Equally significant among the researchers’ findings is the correlation between these tumors and a distinctive mutation signature. In approximately half of the examined tumors, a discernible pattern of mutations emerged, characteristically linked to lesions associated with perturbations in the Apolipoprotein B mRNA-editing enzyme and catalytic polypeptide-like (APOBEC) family of cytidine deaminases. The intrinsic deamination activity of APOBEC enzymes transforms cytosine to uracil during RNA editing. Moreover, compelling evidence posits that a subgroup of APOBEC3 enzymes contributes to an elevated mutation burden in a variety of cancer genomes [10,11]. Notably, ATCs in the present study bearing this mutation signature exhibited a phenomenon known as focal hypermutability, manifesting as localized genomic regions with a higher density of mutations, in contrast to the uniform distribution of mutations across the DNA molecule (Figure 1C) [6]. This intriguing occurrence has been labeled “kataegis”, drawing its inspiration from the Greek term for “thunderstorm”. Given the recent coupling between tumor mutational burden and efficacy of immune checkpoint inhibition in various tumors, the finding of focal hypermutational “hotspots” across the genome may be of potential interest when designing future studies on the role of immunotherapy in ATC patients [12].

Finally, the authors [6] also described non-coding variants that possibly could contribute to tumor development. When specifically investigating promoter and transcription binding sites, 960 SNVs were identified within these regions across 8 ATCs. Apart from conventional *TERT* promoter mutations, no other alterations within regulatory regions were recurrent. However, two cases exhibited unconventional *TERT* promoter variants (Figure 1D). These variants, not present in the database of single nucleotide variants (dbSNP), might potentially impact tumorigenic potential. Interestingly, one of the variants (C168T) was located within the *TERT* promoter, while the other (C105T) is located just upstream of the *TERT* gene’s ATG start site. Further studies of these variants in an additional ATC material is probably warranted.

To summarize, Stenman and colleagues verified that a significant amount of ATC cases harbor deletions in *CDKN2A/B*, with a significant reduction in *CDKN2A/B* mRNA levels. Since deletions of these genes are unusual in WDTCs, this would imply that loss of *CDKN2A/B* marks an important genetic event in the progression of thyroid cancer and possibly opening up interesting approaches for clinical studies using CDK inhibitors. Moreover, the finding of focal hypermutability may be of interest when designing future studies using immune checkpoint inhibition, especially if these results are to be correlated to global tumor mutational burden. Finally, the finding of unconventional variants within the *TERT* regulatory regions may also be of interest, given the correlation between telomerase activity and immortalization.

## Figures and Tables

**Figure 1:**
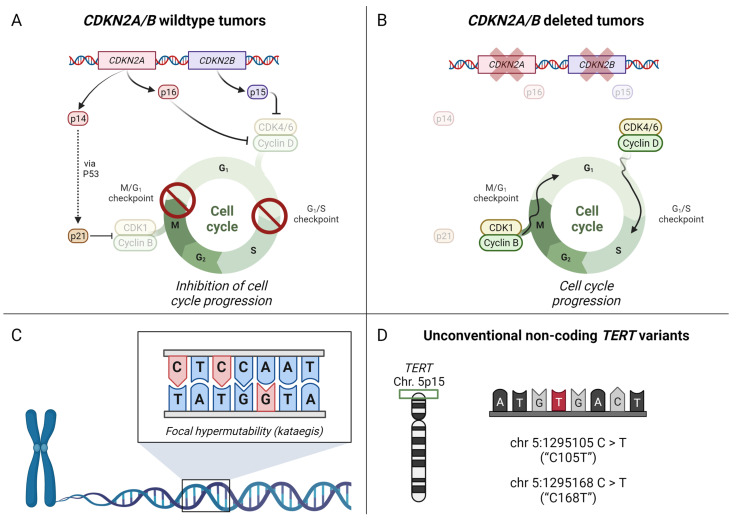
Schematic overview of the main findings in the Stenman paper [6]. By utilizing whole-genome sequencing, the authors corroborate previous findings indicating (**A**,**B**) *CDKN2A/B* deletions in anaplastic thyroid carcinoma (ATC). In *CDKN2A/B* wildtype tumors, expression of p14, p15 and p16 will repress cell cycle progression by inhibition of cyclin-dependent kinase (CDK) interaction with Cyclin B and D. If *CDKN2A/B* is deleted (indicated by red X mark), this inhibition is lifted. (**C**) The authors also identify focal hypermutability (“kataegis”) in tumors with a specific mutational signature coupled to aberrant APOBEC enzyme activity and (**D**) present novel *TERT* promoter variants not previously associated with ATC.
